# Antidiabetic properties of the ethanolic extract of *Rhus coriaria* fruits in rats

**Published:** 2010

**Authors:** S. Mohammadi, S Montasser Kouhsari, A Monavar Feshani

**Affiliations:** Department of Cellular and Molecular Biology, School of Biology, University College of Science, University of Tehran, Tehran, Iran

**Keywords:** Antioxidant, Blood Glucose, α-Glucosidase, Lipids

## Abstract

**Background and the purpose of the study:**

Fruits of *Rhus coriaria L*. (Anacardiaceae) are traditionally used as a table spice in Iran and are highly recommended for diabetic patients.

The purpose of this study was to determine the antidiabetic properties of the ethanolic extract of *Rhus coriaria* fruits and also its mechanisms of action.

**Methods:**

The effects of ethanolic extract of *Rhus coriaria* fruits were measured on blood glucose, lipids and antioxidant enzymes by commercial kits. mRNA levels of insulin (INS) and glucose transporter type-4 (GLUT-4) genes were investigated by RT-PCR (Reverse transcription- polymerase chain reaction) technique. Moreover, its effects on intestinal a-glucosidases was measured using an in vitro method.

**Results and Conclusion:**

Following a single dose administration of the extract it was found that extract could significantly reduce postprandial blood glucose by 24% (at 5 hrs). In the long term experiment, on the day of 21, postprandial blood glucose (PBG) was found to be significantly lower (by 26%) compared to diabetic control group. The plant extract raised markedly serum high-density lipoprotein (HDL) by 34% and also reduced low-density lipoprotein (HDL) by 32%. Also it had noticeable antioxidant effects by elevating superoxide dismutase (SOD) and catalase(CAT) activities by 46% and 77%, respectively. However it did not show a strong effect on glutathione peroxidase (GPX) activity. The extract inhibited maltase and sucrase activities by 44% and 27%, respectively. However it made no changes in the transcript levels of INS and GLUT-4 genes.

It can be concluded that constituents of *Rhus coriaria* fruits have effective components which can be utilized as useful herb for alleviation of diabetes complications.

## INTRODUCTION

Type 2 diabetes is a major public health concern, causing tens of millions of chronic illnesses and a significant number of deaths worldwide each year (1). It is characterized by hyperglycemia and profound alterations in the plasma lipids and lipoproteins profiles (2). Also antioxidant defense system which normally modulates the level of oxidative stress (3) is altered in diabetes (4) and it involves in the pathogenesis of diabetes and its complications (5). Therefore glycemic, lipids profiles and oxidative stress control is fundamental for the management of diabetes (6).

In Iran, *Rhus coriaria* is traditionally used as a table spice especially along with rich dishes and is highly recommended for adjustment of the blood lipids in diabetic patients.

Previous studies on this plant have shown that its fruits (7) and leaves (8) have in vitro antioxidant properties. Also it has already been reported that in vitro hypoglycemic activity of the methanolic extract of fruits of *Rhus coriaria* is due to inhibition of a-amylase (87% inhibition at 50 g/ml) (9).

In this study the antihyperglycemic, hypolipidemic and anioxidant activities of the ethanolic extract of *Rhus coriaria* fruits on diabetic male Wistar rats was investigated in order to verify the use of this herbal medicine in treatment of type 2 diabetes.

## MATERIAL AND METHODS

### 

#### Chemicals and reagents

DEPC Water, Taq polymerase and RNA extraction kit were purchased from CinnaGen (Tehran, Iran). DNase I, RNase free kit was from Fermentas (Ontario, Canada), RT kit and Primers were from Bioneer (Daejon, Korea), dNTPs were from BioFlux (Tokyo, Japan). The kits for determination of glucose, triglyceride (TG), total cholesterol (TC), high density lipoprotein (HDL) were from Chem Enzyme (Tehran, Iran). Hemoglobin Reagent Set was from Ziest Chem (Tehran, Iran), SOD and GPX kits were from Randox (Antrim, UK). All other chemicals and solvents were of the highest commercial grade from Merck (KGaA, Germany) or Sigma (St Louis, MO, USA).

#### Preparation of Rhus coriaria fruits ethanolic extract(RE)

Fruits of *R. Coriaria* were collected from Markazi province of Iran and were authenticated by Professor Ahmad Qahraman and a Voucher specimen (No. 5084) was deposited in the herbarium of University of Tehran (Tehran, Iran). The air dried fruits of *R. coriaria* were finely powdered (400 g) and extracted three times with fresh 96% ethanol at room temperature for 12 hrs. The ethanolic solution after removal of solids was concentrated at 40°C by rotary evaporator and then lyophilized. The percentage yield based on the dried starting material was 16%. The powder was stored at dark at 4^°^C for subsequent experiments.

#### Preparation of Alloxan-induced diabetic wistar rats

Male Wistar rats (*Rattus norvegicus*) weighing 200–250 g were used in this study (Pasteur Institute, Tehran, Iran). Animals were housed six per standard rat cage, in a room with a 12:12 hrs light/dark cycle and controlled temperature (22±1 °C). Diabetes was induced in overnight fasted rats by subcutaneous injection of alloxan monohydrate (100 mg/kg, Sigma, St Louis, MO, USA), dissolved in citrate buffer (pH=4.5), according to a previously described method (10).

### Methods

The rats were divided into five groups of six each. Group I (NC): normal rats were treated with vehicle alone; group II (DC): diabetic rats were treated with vehicle alone; group III (REa+D): diabetic rats were treated with RE at doses of 200 mg/kg BW; group IV (REb+D): diabetic rats were treated with RE at doses of 400 mg/kg BW. Group V (Ac+D in the1st phase or Met+D in the 2nd phase): diabetic rats were treated with acarbose (20 mg/kgBW) or metformin (100mg/kg BW). The doses were selected from pervious publications (11).

In the first phase of this study, following 1, 3, 5, 8 and 24 hrs of oral administration of a single dose of samples to rats, PBG levels were estimated using blood obtained from tail vein and a glucometer (On Call Now, San Diego, USA). After two days, Oral Glucose Tolerance Test (OGTT) was carried out for all rats. In this phase acarbose was used as the reference drug.

In the second phase, two days after OGTT, all rats were administered once a day with samples for 21 days. In this phase metformin was used as the reference drug. PBG levels were estimated at the end of one, two and three weeks of treatments. At the end of treatment period, rats were anesthetized by ether and their bloods were collected. Serum total cholesterol, triglycerides and high density lipoprotein levels and the activities of SOD (EC: 1.15.1.1) and GPX (EC: 1.11.1.9) were measured using commercial kits. Low density lipoprotein and very low density lipoprotein were calculated by formula (12) and CAT (EC: 1.11.1.6) activity was measured by method of Aebi (13).Then the animals were killed and their pancreases and hearts were removed promptly for the estimation of insulin (INS) and glucose transporter-4 (GLUT-4) mRNA expression.

In a separate experiment the inhibitory effects of plant extract on intestinal a-Glucosidases (sucrase and maltase) was measured by an in vitro method.

#### Measurement of RE effects on oral glucose tolerance

NC and DC Groups received distilled water orally. Animals of REa+D and REb+D groups received orally RE, 200 and 400 mg/kg BW respectively.To group Ac+D was given the reference drug, acarbose, (20 mg/kg BW). Thirty min later, each rat received orally a carbohydrate solution (equal proportion of maltose and sucrose) (2 g/kg BW). PBG of each animal was determined at 0 min, just before carbohydrate solution loading, and at 30, 60 and 120 min after that, using a glucometer (On Call Now, San Diego, USA).

#### Gene expression analysis

Pancreas and heart left ventricle tissues of DC, NC and REb+D groups were dissected from rats immediately after sacrificing, placed in liquid nitrogen, and then stored at -70°C until used. Total RNA of tissues were extracted by RNX- Plus kit according to its manual. The DNA free RNA was prepared prior to RT-PCR using DNase I, RNase-free kit. For reverse transcription to obtain cDNA, AccuPower RT PreMix kit, 50 ng/l template RNA and 25 ng/l oligo dT18 were used. The primers were as follows: INS F, 5'-TTCTTCTACACACCCAAG-3'; INS R, 5'- GCAGTAGTTCTCCAGTTG-3' (155-bp) GLUT-4 F, 5'-AGGCACCCTTACCCTTTT-3'; GLUT-4 R 5'-GACAGAAGGGCAACAGAAGC-3' (318-bp); and B-act F, 5'-AGCCATGTACGTAGCCATCC- 3'; B-act R, 5'-TCTCAGCTGTGGTGGTGAAG-3' (248-bp). For PCR reaction, 500 ng of the cDNA was added to a PCR reaction mixture consisting of 10X PCR buffer (2.5 l), 50 MmMgCl2 (0.75 l), 10 mM dNTPs (0.5 l), 10 pM of paired primers (0.5 l of each), 0.25 units of Taq polymerase and distilled water in a total volume of 25 l. The reaction mixture was overlaid in a PCR thermal cycler for 35 cyclic reactions. PCR products were run on 1.5% agarose gels, stained with ethidium bromide and photographed. Images of radiographs were analyzed with TotalLab v1.10 using 1D analysis.

#### In vitro inhibition assay for rat intestinal sucrase and maltase activities

The inhibitory effects of RE on rat intestinal sucrase and maltase activities were determined using a literature method (14).

#### Statistical analyses

All data are presented as means±S.D. for six rats in each group. Comparison between groups and between time points was made by one-way analysis of variance (ANOVA) followed by Duncan's test using SPSS (SPSS Inc, Chicago, USA).

## RESULTS AND DISCUSSION

### 

#### Short-term effects of RE on PBG levels

Administration of the extract at the dose of 400 mg/kg reduced the glucose level at 5 hrs time point compared to both initial level and DC group (Table 1).

**Table 1:** Acute effect of the ethanolic extract of *Rhus coriaria* fruits on postprandial blood glucose levels in Alloxan-diabetics rats.

Groups	Dose (ms/ks BW)	Postprandial blood glucose (mg/dl) Time (h) after a single dose administration of drugs					

		0	1	3	5	8	24
NC	-	69.2±2.3^d^	72±3.5^c^^d^	74.4±3.4^c^^d^	71.2±6.5^c^^d^	64.8±2.9^c^^d^	75.2±4.5^c^^d^
DC	-	334.8±6.3	335±8.6^c^	345.2±7.19^c^	340.2±3.9^c^	343.8±6.9^c^	346±9.9^c^
REa+D	200	338.2±42.2^f^	331.8±11.8^c^^f^	329.6±12.5^c^^f^	316±10.8^b^^e^	346.8±7.6^c^^f^	359.6±6.8^b^^f^
REb+D	400	325.2±5.2^f^	319±6.7^c^^f^	302.8±15.9^c^^d^	289.2±12.3^b^^d^	294.6±21^b^^d^	313.6±13.6^c^^d^
Ac+D	20	318.2±10.2^e^	265.2±7^a^^d^	256.2±7.3^a^^d^	186.2±14.8^a^^d^	199.4±7^a^^d^	266.8±7.8^a^^d^

NC indicates Normal Control; DC, Diabetic Control; RE+D, Diabetic rats treated with ethanolic extract of *Rhus coriaria* fruits Ac+D, Diabetic rats treated with Acarbose.

a p<0.0001

b p<0.007 and

c p>0.096 vs. Time 0

d p<0.0001

e p<0.006 and

f p>0.058 vs. DC.

#### Effects of RE on oral glucose tolerance

As shwon in Table 2, diabetic rats treated with 200 and 400 mg/kg RE showed remarkable decrease in blood glucose at the first hour after carbohydrate solution loading compared to DC group;however they were not as effective as acarbose.

**Table 2:** Effect of the ethanolic extract of *Rhus coriaria* fruits on Oral Glucose Tolerance Test in Alloxan-diabetic rats.

Groups	Dose (mg/kg BW)	Blood Glucose (mg/dl) minutes after administration a single dose of drugs			

		0	30	60	120
NC	-	72.8±2.8^a^	133.8±31.1^a^	126.6±28.5^a^	90.6±6.1^a^
DC	-	157.6±10	347.2±19.9	308.6±12.1	247±9.3
REa+D	200	155.4±8.4^b^	318.4±18.3^b^	257.6±14.7^a^	236.1±12.8^b^
REb+D	400	175±9.7^c^	328.8±13.3^a^	223.2±10.8^a^	232±16^b^
Ac+D	20	154.4±6.8^b^	234.4±10.5^a^	172±6.2^a^	162.8±5.6^a^

NC indicates Normal Control; DC, Diabetic Control; RE+D, Diabetic rats treated with ethanolic extract of *Rhus coriaria* fruits; Ac+D, Diabetic rats treated with Acarbose.

a p<0.0001

b p>0.106 and

c p>0.065 vs. DC.

#### Long-term effects of RE on PBG

The RE at the dose of 400 mg/kg induced significant reductions in PBG levels in diabetic rats, after 7, 14 and 21 days of treatment (Table 3), compared to initial time point. The data of REb+D and Met+D groups were significantly different from those of DC group (p<0.0001).

**Table 3:** Glycemic control by ethanolic extract *of Rhus coriaria* fruits in Alloxan- diabetic rats after 3 weeks treatment.

Groups	Dose (mg/kg BW)	Postprandial blood glucose (mg/dl) Days after single dose treatment daily			

		0	7	14	21
NC	-	76.8±2.8^d^	82±5^c^^d^	77±6^c^^d^	83.5±5^c^^d^
DC	-	324.8±7.2	347±10.3^b^	364±8.1^a^	353.3±11.1^b^
REa+D	200	337.4±15.4^f^	319.2±11.9^c^^f^	311.6±19.4^c^^e^	314.7±22.7^c^^f^
REb+D	400	331.4±19.6^f^	282.2±17.7^b^^d^	269.8±16.3^a^^d^	261.6±22.4^a^^d^
Met+D	100	318.2±10.2^f^	251.3±6.7^b^^d^	213.1±5.9^a^^d^	190.2±8.2^a^^d^

NC indicates Normal Control; DC, Diabetic Control; RE+D, Diabetic rats treated with ethanolic extract of *Rhus coriaria* fruits; Met+D, Diabetic rats treated with Metformin.

a p<0.0001

b p<0.004 and

c p>0.141 vs. Time 0

d p<0.0001

e p<0.001 and

f p>0.065 vs. DC.

#### Effects of RE on rat intestinal sucrase and maltase activities

The crude ethanolic extract of *Rhus coriaria* fruits showed strong inhibitory effects for both sucrase and maltase activities,25.38%(±2.05) and 44.33%(±1.14) respectively.

#### Effects of RE on INS and GLUT-4 genes expression

Densitometric scanning revealed no increase in INS (p>0.156) and GLUT-4 (p>0.564) transcripts by RE, as compared to DC group.

#### Effects of RE on blood lipids

Treatment with RE (400 mg/kg) strongly increased (Table 4) the level of HDL, in a way that it was comparable to NC rats (p>0.988). Also it reduced the level of LDL significantly (33%, p<0.012) compared to DC group.

**Table 4:** Effects of the ethanolic extract of *Rhus coriaria* fruit on plasma lipids of Alloxan-diabetic rats, after 21 days of treatment.

Groups	Dose (mg/kg BW)	Serum lipids (mg/dl)								

		TG	TC	HDL	LDL	VLDL
NC	-	82.2±5.6^a^	81.4±2.8^a^	45.8±3.2^b^^e^	19.2±4^a^	16.4±1.1^a^
DC	-	144.2±5.3	112.6±9	35±5.6	48.8±13.8	28.8±1
REa+D	200	141.6±7.5^d^	111.7±4.8^d^	40.8±8.3^d^	42.6±9.51^d^	28.3±1.5^d^
Met+D	100	131.4±3.3^c^	102.1±6.7^d^	37.1±3.7^d^	38.3±9.8^d^	26.3±0.6^d^

DNC indicates Normal Control; DC, Diabetic Control; RE+D, Diabetic rats treated with ethanolic extract of *Rhus coriaria* fruits ; Met+D, Diabetic rats treated with Metformin.

a p<0.0001

b p<0.001

c p<0.031 and

d p>0.086 vs. DC

e p>0.988 vs. NC.

#### Effects of RE on antioxidant enzymes activities

As presented in Table 5, the RE-treated groups showed no remarkable change in GPX activity, however a strong increase was observed in SOD and CAT activities at the dose of 400 mg/kg compared to DC group.

**Table 5:** Changes in the activities of superoxide dismutase, glutathione peroxidase and catalase of erythrocytes of Alloxan-diabetic rats made ethanolic extract of *Rhus coriaria* fruits.

Groups	Dose (mg/kg BW)	Blood antioxidant enzymes		

		SOD (U/g Hb)	GPX (U/g Hb)	CAT (K/g Hb)
NC	-	2978±95.2^a^	55.67±2.21^a^	1.441±0.09^a^
DC	-	1916.8±22.1	31.91±5.54	0.508±0.031
REa+D	200	2455.7±83.4^a^	30.84±6.62	0.755±0.053^a^
REb+D	400	2793.8±90.2^a^	35.45±1.7^c^	0.901±0.069^a^
Met+D	100	2048.1±19.8^c^	39.33±4.59^b^	0.533±0.034^c^

NC indicates Normal Control; DC, Diabetic Control; RE+D, Diabetic rats treated with ethanolic extract of *Rhus coriaria* fruits; Met+D, Diabetic rats treated with Metformin.

a p<0.0001

b p<0.004 and

c p>0.107 vs. DC

## DISCUSSION

Previous findings demonstrated that some genus of *Rhus* like *Rhus chirindensis* (15) and *Rhus chinensis* (16) have hypoglycemic properties.

In the Alloxan-induced diabetic rats, administration of the extract of *Rhus coriaria* fruits produced a statistically significant acute and long-term decrease in postprandial blood glucose concentration, which were not comparable to the effects of the reference drugs. It has already been reported that *rhus coriaria* might have hypoglycemic activity by inhibition of alpha-amylase, a glycoside hydrolase (9).Results of this study showed no change in the INS and GLUT- 4 genes expression, so antihyperglycemic effects of *Rhus coriaria* fruits may be related to modulation of insulin secretion or action (17). OGTT results and the ability of inhibiting the a-glucosidases activities by the extract indicate that control of postprandial glucose level might be mediated through the inhibition of carbohydrate digestion or absorption (16).

**Figure 1:**
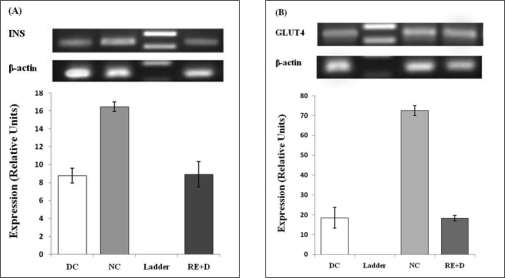
Changes in insulin and cardiac glucose transporter-4 mRNA expression profile in rats. (A) Analysis of INS transcripts (186 bp) in pancreas tissue in RE treated diabetic rats showed no elevated levels of INS transcripts compared with DC rats (p>0.156). (B) Analysis of GLUT-4 transcripts (449 bp) in heart tissue in RE treated diabetic rats showed no elevated levels of GLUT-4 transcripts compared with DC rats (p>0.564). The data represent the average of three or four samples (only one image is shown). NC: Normal Control; DC: Diabetic Control; RE+D: Diabetic rats treated with *Rhus coriaria* fruits ethanolic extract.

Oral administration of ethanolic extract of *Rhus coriaria* fruits increased SOD and CAT levels of the red blood cells. This finding upholds the in vitro antioxidant activity of *Rhus coriaria* demonstrated perviously (7). Antioxidant activity of *Rhus coriaria* could also be useful for prevention of diabetes complications due to hyperglycemia (11)Figure 1.

This study aside from *Rhus coriaria* antihyper- glycemic and antioxidant activities indicated that extract may also reverse dyslipidemia associated with diabetes by increasing the HDL level and decreasing the LDL level similar to some other herbs previously described (18).

In conclusion, the use of *Rhus coriari*s*a* in Iranian folk medicine as a hypolipidemic agent has a significant correlation with the scientific data generated in this study. Also the present study explains two other properties of *Rhus coriaria* fruits on diabetic rats which can improve the life of type 2 diabetic patients: mild antihyperglycemic and potent antioxidant peroperties. Further studies are required to show the active constituent(s) of *Rhus coriaria*.
